# PW03-029 – Risk factors for AA-type amyloidosis in Germany

**DOI:** 10.1186/1546-0096-11-S1-A255

**Published:** 2013-11-08

**Authors:** N Blank, P Lohse, U Hegenbart, H-M Lorenz, SO Schoenland

**Affiliations:** 1 Hematology, Oncology and Rheumatology, University of Heidelberg, Heidelberg, Germany; 2 Institute of Laboratory Medicine and Human Genetics, Singen, Germany

## Introduction

Patients with autoinflammatory diseases or chronically active autoimmune disease are at risk for developing AA-type amyloidosis (AA).

## Objectives

To identify risk factors for AA in patients with familial Mediterranean fever and chronic inflammatory autoimmune diseases living in Germany.

## Methods

Seventy-three patients with FMF and 39 patients with active systemic inflammation due to autoimmune or idiopathic inflammation were evaluated in our reference center for amyloidosis. Mutations in MEFV and the SAA-1 exon 3 variants were analyzed for their contribution to AA risk. Amyloidosis was detected in all cases by congo red staining of kidney or bowel biopsy specimens and AA was classified by immunohistochemistry.

## Results

Thirteen patients with FMF (18%) had AA which was diagnosed either before their first contact or during the initial presentation in our center. The relative risk (RR) for amyloidosis of two M694V mutations was 1.15, compared to a RR of 1.00 for a single M694V substitution and a RR of 0.46 in the absence of a M694V mutation in these patients. However, a homozygous SAA 1.1 genotype had a RR of 3.65 (p=0.003), compared to a SAA RR of 0.63 for SAA 1.1+other and to a RR of 0.32 for SAA other+other. FMF patients with AA were older at FMF diagnosis (31 vs 23 years, p=0.003), and AA was diagnosed at a median age of 34 years (95%CI 28.6-47.3). The delay of FMF diagnosis was 17 years in patients with AA and 9 years in patients without (p=0.064).

Twenty-three patients with rheumatic diseases or idiopathic inflammation had AA. A control group of 16 patients with chronically active systemic inflammation was recruited from consecutive patients in our rheumatology department. Twenty-two of 23 patients with AA (96%) had the SAA1.1+1.1 genotype compared to 5 of 16 patients (31%) in the control group, resulting in a RR of 2.05 for AA (p=0.054). Within this group, 7 patients with idiopathic inflammation could not be associated with any autoimmune or autoinflammatory disorder and were considered to be idiopathic AA. These patients with idiopathic AA were older at the onset of inflammation (51.0 vs 25.5 years, p=0.17) and were older at AA diagnosis (63.0 vs 53.5 years, p=0.9), but had a shorter duration of inflammation to AA diagnosis (16.0 vs 20.0 years, p=0.19).

## Conclusion

Our data show that the SAA1.1+1.1 genotype is associated with a higher risk for AA than homozygosity for the pyrin M694V mutation. Although M694V+M694V is associated with a more severe disease course, our data show that SAA1.1+1.1 is the major risk factor for AA. In patients with chronically rheumatic diseases or idiopathic AA, homozygosity for SAA1.1 carries a RR of 3.65. Determination of the SAA polymorphism could be useful to assess the risk for AA in patients with chronic inflammatory conditions.

## Disclosure of interest

None declared

